# Multimodal Sequencing and Reanalysis Approaches to End the Diagnostic Odyssey of Individuals with Suspected Rare Monogenic Diseases

**DOI:** 10.3390/genes17060647

**Published:** 2026-05-31

**Authors:** Catherine A. Brownstein, Jill A. Madden, Wanqing Shao, Casie A. Genetti, Jason Chin, Vincent D. Ustach, Monica H. Wojcik, Anna Madden, Nathaniel Edisis, Heng Li, Daniel A. Johnson, Kirsty McWalter, Jessica Noya, Klaus Schmitz-Abe, Shira Rockowitz, Pankaj B. Agrawal, Scott Newman, Joseph M. Devaney, Paul Kruszka, Alan H. Beggs

**Affiliations:** 1 The Manton Center for Orphan Disease Research, Division of Genetics and Genomics, Boston Children’s Hospital, Harvard Medical School, Boston, MA 02115, USAnateedisis@gmail.com (N.E.); klaus.schmitz-abe@childrens.harvard.edu (K.S.-A.); beggs@enders.tch.harvard.edu (A.H.B.); 2 Children’s Rare Disease Collaborative, Boston Children’s Hospital, Boston, MA 02115, USA; 3 GeneDx, LLC, Gaithersburg, MD 20877, USA; 4 Department of Data Science, Dana-Farber Cancer Institute, Harvard Medical School, Boston, MA 02215, USA; 5 Division of Neonatology, Department of Pediatrics, University of Miami Miller School of Medicine, Holtz Children’s Hospital, Jackson Health System, Miami, FL 33136, USA; 6 Division of Pediatric Genetics, University of Virginia School of Medicine, Charlottesville, VA 22903, USA

**Keywords:** long-read sequencing, RNA-seq, iterative reanalysis, diagnostic odyssey, rare disease, multimodal genomics, variant interpretation

## Abstract

Background/Objectives: Genomic testing has transformed rare-disease diagnostics, yet a substantial proportion of individuals remain without a molecular diagnosis even after short-read exome sequencing (SR-ES) or short-read genome sequencing (SR-GS) and repeated conventional analysis. Methods: To address this persistent gap, we evaluated a coordinated multimodal reanalysis framework for deeply investigated families with suspected monogenic disease. Six families (20 individuals; 8 affected individuals) that had remained unsolved after prior comprehensive testing were reviewed prospectively in weekly interdisciplinary case conferences over one year. Available data included SR-ES, SR-GS, long-read genome sequencing (LR-GS), RNA-seq, optical genome mapping, mobile-element analysis, and mitochondrial genome analysis. The goal was not to test a single modality in isolation, but to assess whether systematic escalation across complementary assays plus continued reinterpretation could improve case resolution. Results: Three families (50%) achieved a reportable molecular diagnosis, two (33%) yielded strong candidate findings requiring additional evidence, and one (17%) remained without a definitive new molecular diagnosis, although reinterpretation of a previously identified *NOTCH3* variant provided a possible partial explanation. Resolved cases included compound-heterozygous variants in *KLHL40*, a 119 kb multi-exon deletion in *TTN*, and a recurrent insertion in *RNU4-2*. Candidate findings included biallelic *NARS2* variants and a 1.3 kb intragenic deletion involving *ZEB2*. Functional transcriptomic analyses supported the *KLHL40* and *TTN* diagnoses but did not demonstrate a splicing consequence for the candidate *NARS2* intronic variant in cardiac tissue. Conclusions: This small pilot cohort is not intended to estimate general diagnostic yield, but it demonstrates that a coordinated multimodal framework can reveal different sources of added value, including structural variant discovery, orthogonal functional support, and reinterpretation of existing short-read data as knowledge evolves. These findings underscore that archived short-read exome and genome data can retain substantial diagnostic value years after initial testing, particularly when reanalyzed with updated pipelines, expanded disease gene knowledge, and orthogonal multimodal evidence. Adoption of iterative, team-based multimodal strategies may help resolve the most complex unsolved rare-disease cases.

## 1. Introduction

The advent of genomic testing has transformed the landscape of rare disease diagnosis, enabling timely and precise identification of genetic variants. This transformation is fueled by the rapid pace of rare disease gene discovery, substantial reductions in the cost of sequencing technologies including short-read exome sequencing (SR-ES) and short-read genome sequencing (SR-GS), and significant investments aimed at translating genomic research into clinical practice. As a result, ES and GS have become powerful tools in uncovering genetic causes for a wide range of conditions, significantly improving diagnostic rates and patient care.

Despite these advancements, a substantial proportion of individuals remain undiagnosed even after comprehensive genetic testing. Diagnostic yields of SR-ES range between 25 and 58% [1], with SR-GS contributing an additional yield of up to 8% in unresolved cases [2,3]. These undiagnosed cases often involve individuals who have undergone extensive evaluations by multiple specialists and detailed clinical investigations, exemplifying the challenges inherent in diagnosing complex or atypical presentations of genetic disorders. For such individuals, the search for answers can become a protracted and frustrating diagnostic odyssey, underscoring the need for novel approaches to resolve these cases.

The quest to improve diagnostic outcomes has spurred the development and application of emerging genomic technologies. Transcriptome sequencing, for instance, has demonstrated its potential to increase diagnostic rates by uncovering transcriptional changes associated with pathogenic variants. Identifying functional consequences of variants can increase diagnostic rates through iterative reanalysis [1]. However, its routine use is limited by cost and logistical challenges, including tissue-specific gene expression.

Similarly, technologies such as long-read sequencing and long-range transcriptomics hold promise for resolving difficult-to-detect variants, including structural variants and repeat expansions. Recent efforts have emphasized the potential of long-read sequencing to detect complex genomic variation [4], and integration into clinical workflows [5]. Finally, comprehensive and continued reanalysis of exome data is beneficial for undiagnosed individuals with likely Mendelian conditions [6].

Importantly, previously generated short-read exome or genome data remain a valuable resource even years after initial analysis. As variant-calling methods, annotation resources, gene-disease associations, and phenotype-matching tools improve, reanalysis of existing data can identify diagnoses that were not technically or interpretively accessible at the time of the original test. The availability of complementary multimodal data can further increase diagnostic power by providing orthogonal evidence for candidate variants, clarifying functional consequences, and revealing classes of variation that may be difficult to detect or interpret from short-read data alone.

Together, these studies reinforce the conclusion that reanalysis, long-read sequencing, and RNA-based assays can each expand the capacity to detect pathogenic variants. However, most prior reports evaluate these modalities separately or retrospectively. Less well described is how to organize them prospectively within a single real-world workflow for deeply investigated families, including when reanalysis of existing short-read data is sufficient and when to escalate to LR-GS, RNA-seq, OGM, mitochondrial analysis, or other complementary assays. To address this gap, we examined a small pilot cohort of six families with suspected monogenic disease that remained unsolved despite extensive prior testing. Our aim was not to argue that any one modality is universally superior to short-read reanalysis, but rather to describe the added value of a coordinated multimodal framework that combines iterative reinterpretation, phenotype re-review, and targeted deployment of complementary assays.

## 2. Methods

Families (n = 6, 4 trios and 2 quads; Table 1) were selected on the basis of having a suspected Mendelian diagnosis and prior negative SR-ES or SR-GS (Supplemental Figure S1). A suitable family structure to allow segregation analysis was required. The exclusion criterion was suspicion of a non-monogenic cause. Two families had more than one affected individual (MAN_0146 and MAN_2064), BOS_1210 included an unaffected sibling, and the remaining families were simplex. Simplified pedigrees are shown in Supplemental Figure S2. Four families had previously extracted and stored DNA (−20 °C), while samples from the remaining two families (MAN_2064 and BOS_1596) were whole blood stored frozen (−20 °C) in EDTA tubes (Table 2). One individual had previously extracted RNA isolated from cardiac muscle (MAN_0146-04), and two individuals had previously extracted RNA from skeletal muscle (BOS_1596-01 and BOS_1210-01) (Table 2).

### 2.1. Ethics Declaration

This study adhered to the ethical principles stipulated in the Declaration of Helsinki of the World Medical Association. The study protocol and all associated procedures received approval from the Boston Children’s Hospital Institutional Review Board (10-02-0053 and 03-08-128R). Informed consent was obtained from all participants.

### 2.2. Case Summaries

BOS_1596-01 is a 4 yo male with a histopathological diagnosis of nemaline myopathy from muscle biopsy at 4mo of age. Pregnancy history was reportedly normal. At birth the proband presented with mild hypotonia, head lag and feeding difficulties with concerns for aspiration and weight gain. He received the majority of nutrition by g-tube until age 2 yo. History is notable for gross motor delays and proximal and facial muscle weakness without significant respiratory concerns. The proband was able to walk with support at 2 yo. The proband is an only child with healthy parents and no family history of neuromuscular disease.

Prior testing for the proband included clinical SR-ES (in 2021), which did not identify any reported variants. A comprehensive neuromuscular gene panel was also inconclusive (2021) but did identify a heterozygous pathogenic variant in an autosomal recessive (AR) gene: a heterozygous pathogenic *RYR1* frameshift mutation, chr19-38504810 G>GC, NM_000540.3:c.8136dup (p.Asp2713Argfs*10), inherited from the healthy father. This finding is consistent with the recessive nature of the variant, as frameshift mutations in *RYR1* are generally inherited in a recessive fashion and are not typically associated with dominant cases. Supporting this, population data from gnomAD reveals 97 heterozygous loss-of-function (LOF) *RYR1* variants and a pLI of 0, indicating tolerance for frameshift mutations in the heterozygous state. Additionally, a variant of uncertain significance (VUS) chr3-42686291 C>T, NM_152393.4:c.673C>T (p.Arg225Cys) in *KLHL40* was identified, also inherited from the father. Muscle biopsy tissue was available for RNA sequencing. DNA and blood samples have been stored for all family members. Analysis showed no evidence of consanguinity, with a reported rate of 0.45%.

BOS_1210-01 is a 20 yo male with a histopathological diagnosis of congenital fiber-type disproportion (CFTD) from muscle biopsy at age 5. Pregnancy history was reportedly normal other than reduced fetal movement. At birth the proband presented with bilateral club feet and generalized hypotonia leading to a clinical diagnosis of arthrogryposis multiplex congenita. As of 17 yo he was fully ambulant; however, he has experienced progressive mobility issues in his knee, ankle, hip, jaw, and shoulder joints. Pulmonary function was subnormal, but respiratory support was not required. Cardiac status was normal at last evaluation and there is no family history of neuromuscular disease.

The proband’s healthy sister and both healthy parents are enrolled in the study, with DNA samples stored for all family members. Biological parentage was confirmed and there was no evidence of consanguinity. Short-read exome (2019), genome (2022) and RNA (2022) sequencing to diagnose this case was recently reported [7]. Initial read depth analysis of next generation panel testing for 43 neuromuscular disease genes suggested the presence of copy number loss of *TTN* gene exons encoding portions of the fibronectin type II repeats in the A-band region of titin. Subsequent short-read research-based ES failed to identify this or any other potentially pathogenic variants in *TTN*, but ddPCR using probes from the A-band region confirmed an apparent loss of one copy in the proband and apparently two normal copies in the parents.

MAN_1875-01 is a 19 yo male with a complex multisystem medical history with no unifying diagnosis, most notable for an impression of potential lipodystrophy. He presented originally at 10 yo with progressive demyelinating polyneuropathy. Additional features include hepatic steatosis, hepatosplenomegaly, lymphatic dysfunction and lymphedema, painful subcutaneous nodules, progressive weakness and fatigue, stable restrictive lung disease, recurrent infections, optic nerve neuritis, and papillary thyroid cancer, status post removal. He has normal cognition and no contributory family history.

Testing history for this individual and family included SR-GS and Deletion/Duplication Analysis (2021), which identified a heterozygous pathogenic variant in ANO5, chr11-22274605 C>T NM_213599.3:c.2272C>T (p.Arg758Cys) and a maternally inherited variant of uncertain significance in ANO5, chr11-22211323 C>T NM_213599.3:c.138+9C>T. This was confirmed by the Comprehensive Neuromuscular Diseases NGS Gene Sequencing Panel (2017). However, the ANO5 variants are considered unlikely to explain the clinical phenotype. First, the individual’s presentation is inconsistent with ANO5-related pathology, which typically manifests as a muscle disorder or gnathodiaphyseal dysplasia in dominant forms. Instead, this individual’s clinical picture strongly supports a neuropathic rather than myopathic origin. Specifically, multiple electromyography (EMG) evaluations demonstrate generalized axonal and demyelinating sensorimotor polyneuropathy. Creatine kinase (CK) levels remain within normal limits, further arguing against a primary muscular involvement. Second, the ANO5 splice-region variant (c.138+9C>T) is predicted to have minimal functional impact. A heterozygous variant of uncertain significance in FLT4, chr5-180614189 C>T, NM_182925.5:c.3220-10G>A was also identified via SR-GS. SR-ES trio (2018) was otherwise negative. Charcot–Marie–Tooth comprehensive neuropathy panel (seq, del/dup; 2017) was also negative. Kennedy’s disease gene testing (2018) was negative for the CAG repeat expansion.

GeneDx reanalysis of SR-ES (2021) identified a de novo *NOTCH3* variant (chr19-15170764 A>G, NM_000435.3:c.4798T>C, p.Cys1600Arg) categorized as a VUS. At the time of reporting, variants in *NOTCH3* were principally associated with CADASIL, which was not felt to align with the overall phenotype. No cerebrovascular manifestations suggestive of classic CADASIL were documented in the available clinical records reviewed for this study.

MAN_0009-01 is a 29 yo male with a complex medical history suggestive of a congenital syndrome. He was delivered at term via cesarean section due to prolonged labor, but the pregnancy and delivery, otherwise, were uncomplicated. He has significant global developmental delay; he is currently non-verbal and unresponsive to commands. He began walking at age 7 and is now ambulatory but often needs support. He has been seen by ophthalmology for visual impairment, nystagmus, right retinal detachment, right esotropia, and night blindness. He also has bilateral sensorineural hearing loss and wears hearing aids. He has a recent history of chronic kidney disease with hypertension. Other features include abnormal movements, craniofacial dysmorphism, microcephaly, short stature, poor weight gain, chest deformities, lordosis, osteoporosis, and dental caries. There is no relevant family history.

Prior negative testing included research trio SR-ES (2017), chromosomal microarray (2008), and Fragile X testing (2009).

MAN_0146-01 and MAN_0146-04 are two affected and deceased female neonates with hypertrophic cardiomyopathy, severe pulmonary hypertension, and hypotonia. The older sister, MAN_0146-01, was full term and born via vacuum-assisted vaginal delivery. The pregnancy was notable for intrauterine growth retardation, along with pericardial effusion and cardiomegaly on ultrasound. Fetal tachycardia was noted prior to delivery. At 5 min of life, she became limp and cyanotic with poor perfusion. She was transferred to the NICU with profound metabolic acidosis and respiratory distress requiring intubation. An echocardiogram showed severe biventricular hypertrophy and severe right ventricular and pulmonary artery hypertension. She passed away at 3 weeks. The younger sister, MAN_0146-04, similarly appeared apneic and cyanotic at birth requiring intubation, and she was found to have severe lactic acidosis and right ventricular hypertrophy. The mother was induced at 38 weeks due to diminished diastolic flow. Fetal ultrasound, echocardiogram, and MRI were normal. Postnatal echocardiogram showed declining left ventricular function as well as systemic pulmonary hypertension. She passed away at 1 week of age. There is no additional relevant family history.

Research testing for MAN_0146-01 included trio SR-ES and singleton SR-ES performed at two separate laboratories in 2012 and 2014, as well as quad SR-GS (2023) and RNA sequencing (RNA-seq, 2015) on cardiac muscle. Analysis revealed no evidence of consanguinity or identical-by-descent, with a reported rate of 0%.

MAN_2064-01 and MAN_2064-04 are two affected brothers with a shared presentation of a neurodevelopmental disorder. MAN_2064-01 is 52 years old and MAN_2064-04 is 48 years old. They were born after unremarkable pregnancies and vaginal deliveries and both have a history of hypospadias status post repair. The brothers have a history of developmental delay and moderate intellectual disability, joint hypermobility, and keratoconus with associated progressive vision problems over the last few decades. Behavioral concerns include obsessive–compulsive disorder and Tourette syndrome. Both individuals exhibit subtle facial differences including a long face with prominent jaw, dense eyebrows, and down-slanting eyes, as well as pectus excavatum. There is no additional contributory family history.

Prior genetic testing for the probands included research-based quad SR-ES (2020), which yielded negative results. Additional testing included Fragile X syndrome analysis (1992), which was negative, and karyotype analysis (1978), confirming a 46, XY chromosomal complement.

### 2.3. Technologies Used

#### 2.3.1. Sequencing and Variant Calling

SR-GS data were generated and processed through a DRAGEN Bio-IT Platform and VExP6. LR-GS data were generated on PacBio platforms and analyzed with the PacBio human whole-genome workflow, with custom integration into Genuity Sequence Miner. De novo LR-GS assemblies generated with hifiasm were compared with the draft human pangenome by PGR-TK, and structural variants (SVs) and copy number variants (CNVs) of interest were further interrogated with a k-mer counting approach. PacBio Revio HiFi sequencing achieved mean coverage of approximately 25–30× with median read lengths of 15–18 kb. HiFi reads were assembled with hifiasm v0.18.9 and SVs were called with PBSV v2.8. Optical genome mapping (OGM) was performed on the Bionano Saphyr platform using a minimum molecule-length cutoff of 150 kb, effective coverage >100×, and rare-variant and de novo analysis pipelines in Bionano Access v1.8.1. RNA-seq used poly(A)-enriched stranded libraries sequenced on Illumina NovaSeq (~100 million paired-end reads per sample), aligned with STAR v2.7, and analyzed for differential expression and splicing using LeafCutter v0.2.9 and MAJIQ v2.5. Mobile-element insertions (Alu, HERV, LINE1, and SVA) were detected with xTea v0.1 [8]. Mitochondrial genome analysis was performed by GeneDx.

#### 2.3.2. Variant Prioritization and Interpretation

**Figure 1:**
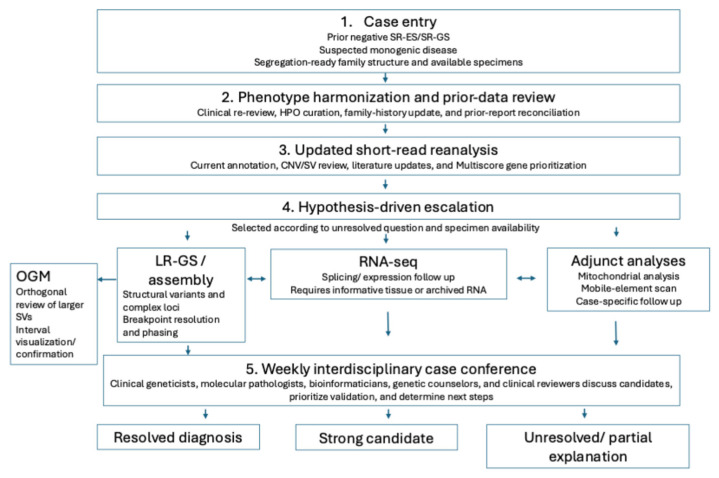
Overview of the coordinated multimodal diagnostic workflow. Families entered the framework after negative SR-ES/SR-GS and underwent phenotype harmonization, updated short-read reanalysis, hypothesis-driven escalation to complementary assays based on the unresolved question and available specimens, and weekly interdisciplinary review. Outcomes were adjudicated as resolved, strong candidate, or unresolved/partial explanation.

Variants were filtered for population frequency <0.001 in gnomAD (v4.1.1) and then prioritized using phenotype relevance, inheritance, variant class, and computational evidence. GeneDx Multiscore was the AI-assisted phenotypic gene-ranking tool used in this workflow [9]; additional in silico evidence included CADD v1.7 [10,11], REVEL v1.3 [12], SpliceAI v1.3.1 [13], and Pangolin v1.0.1 [14]. No single hard CADD threshold (or other in silico cutoff) was used as an exclusion criterion. Instead, these scores were interpreted as supporting evidence in the context of gene-disease validity, segregation, phenotype concordance, and orthogonal data. The workflow followed the escalation logic summarized in Figure 1: re-review phenotype data and prior SR-ES/SR-GS results; prioritize unresolved hypotheses for additional assays based on variant type and specimen availability (for example, LR-GS for suspected structural variation or difficult genomic regions, RNA-seq for predicted splicing/expression effects in informative or available tissue, OGM for orthogonal interrogation of larger SVs, and mitochondrial analysis when clinically indicated); and review ordered candidates in weekly interdisciplinary meetings of clinical geneticists, molecular pathologists, bioinformaticians, and genetic counselors. Manual review and orthogonal confirmation were required before a structural or complex variant was considered reportable. Classification followed ACMG/AMP guidance when applicable, and cases were adjudicated by consensus as resolved, strong candidate, or unresolved.

Multiscore was applied to all eight probands as a prioritization tool rather than a diagnostic classifier. Inputs included HPO terms, free-text clinical summaries, and a predefined gene list. Output was a prioritized list of genes, scored by percentile rank across the active reference set, with 100% assigned to the top-ranked gene and a rank of (1/n_genes) × 100% assigned to the bottom-ranked gene. Variants identified by short-read or long-read pipelines were joined on gene annotation, and the resulting gene ranks were used to focus manual review. Multiscore ranks were not used in isolation to classify variants, and low-ranking genes were not automatically excluded when orthogonal genetic or functional evidence supported review.

Multiscore uses gene-phenotype annotations from OMIM, the literature, and GeneDx SR-ES/SR-GS-positive monogenic cases, which are updated on a monthly cadence. For each Multiscore test, the Multiscore Data Reference (MDR) records the OMIM release, the literature date cutoff, and GeneDx case cutoff. Two MDRs were used in this study (Table 3).

A consistent background gene list was analyzed for each case within a given MDR so that rankings were not biased by the genotypic profile of any specific proband. This is a modification from a previous study [9], in which only genes with variants passing genotype filters were scored. An important limitation is that genes absent from a given MDR cannot be prioritized in that analysis, and ranks generated under MDR1 and MDR2 should therefore be interpreted in the context of their respective gene lists.

MDR1 leverages literature published by 31 December 2023, GeneDx cases closed by 31 December 2023, OMIM release 3 September 2023 (https://github.com/obophenotype/human-phenotype-ontology/releases/tag/v2023-09-01 (accessed 5 November 2025)). The gene list for MDR1 comprised all genes associated with an mRNA transcript in GeneDx’s database as of 1 August 2023 (n = 19,215).

MDR2 leverages literature published by 30 September 2025, GeneDx cases closed by 30 September 2025, OMIM release 1 September 2025 (https://github.com/obophenotype/human-phenotype-ontology/releases/tag/v2025-09-01 (accessed 5 November 2025)). The gene list for MDR2 comprised the union of three sets of genes collected on 28 August 2025: all genes associated with an mRNA transcript in GeneDx’s database (n = 19,220), all genes with validated or candidate diseases in GeneDx’s database (n = 3818), and a supplement of non-coding genes identified by GeneDx’s clinical team (n = 41). The number of genes in the union of these three sets is n = 19,260.

All eight cases were analyzed in Multiscore using MDR1 in 2024. Because *RNU4-2* was absent from the MDR1 gene list and present in the MDR2 gene list, MAN_0009 was reanalyzed in Multiscore using MDR2 in 2025.

## 3. Results

After one year of weekly meetings, three families were adjudicated as resolved at a reportable molecular level; two yielded strong candidate findings that remained below a definitive diagnostic threshold, and one remained unresolved without a definitive new molecular diagnosis (Table 4 and Table 5). In the unresolved case, reinterpretation of a previously identified variant provided a possible partial explanation but was insufficient to classify the family as solved.

BOS_1596: LR-GS initially suggested a candidate NEB deletion. Because the call required orthogonal confirmation, DNA was additionally analyzed by OGM to visualize the interval directly across the suspected breakpoints. The deletion was not confirmed and was reclassified as a false-positive LR-GS finding, illustrating the need for orthogonal validation of structural calls in complex loci.

Proband-only clinical ES had previously identified a heterozygous variant in *KLHL40*, chr3-42686291 C>T, NM_152393.4:c.673C>T, p.(Arg225Cys), a gene associated with recessive nemaline myopathy. As only one variant had been identified, it was not highlighted as being causative of the individual’s phenotype.

**Figure 2:**
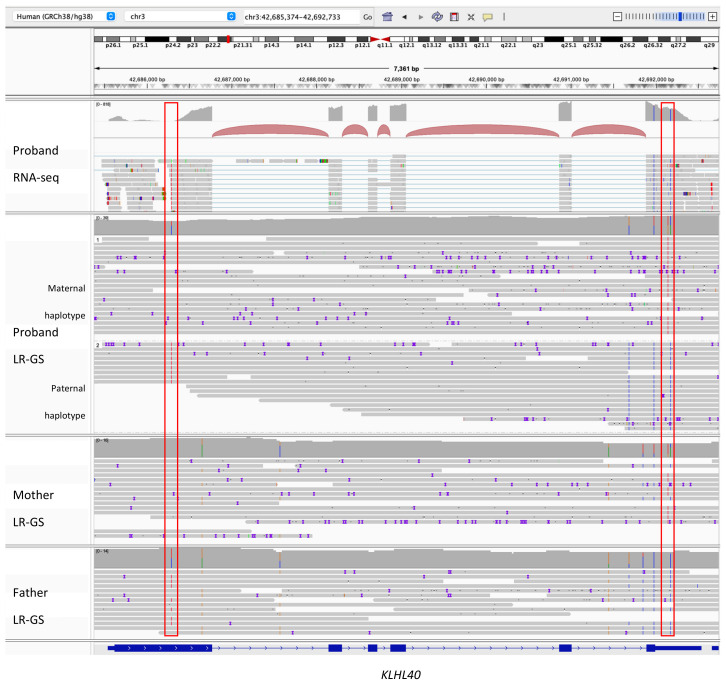
Diagnosis of *KLHL40*-related nemaline myopathy by combined LR-GS and RNA-seq analysis. Integrated Genomics Viewer analysis of the *KLHL40* locus in family BOS_1596. LR-GS in bottom three panels confirmed the presence of a paternal *KLHL40* missense variant, NM_152393.4:c.673C>T, p.(Arg225Cys), in exon 1 (red vertical line representing a “T” base in reads from the paternal allele within red box at left), and demonstrated apparently normal overall structure of the gene with no evidence of gross rearrangements or other structural variants. LR-GS also identified a rare maternal variant in the 3′ UTR, NM_152393.4:c.Ter152G>T, previously unrecognized by clinical SR-ES and panel testing. RNA-seq analysis of the proband’s skeletal muscle biopsy (top panel) revealed normal splicing pattern but demonstrated near complete mono-allelic expression of the paternal transcript as only one read of 863 included the maternal c.Ter152G>T variant (the red vertical line representing a “T” base in maternal reads highlighted in the red box at right).

Trio research SR-GS (Sema4, 2022) identified a maternally inherited *KLHL40* 3′ UTR variant, chr3-42692145G>T, NM_152393.4:c.Ter152G>T, not previously reported by the clinical ES or panel testing and demonstrated paternal inheritance of the p.Arg225Cys missense variant. LR-GS confirmed the presence of these two variants and their inheritance patterns, and demonstrated apparently normal overall structure of the gene with no evidence of gross rearrangements or other structural variation (Figure 2). Review of the proband’s *RYR1* gene also failed to find evidence for a second hit on the maternal allele, focusing further analysis on *KLHL40*. RNA-seq analysis of the proband’s skeletal muscle biopsy revealed normal *KLHL40* splicing but demonstrated near complete mono-allelic expression of the paternal transcript carrying the p.Arg225Cys variant, as only one read of 863 included the maternal c.Ter152G>T variant. Subsequent review of the literature revealed that this 3′ UTR variant had been recently reported as splice altering [15] providing an explanation for clinical impact of the heterozygous paternal missense variant in the proband. Multiscore ranked the phenotype match between the proband and *KLHL40* in the top 0.4% of genes tested (rank = 99.6%). This family was therefore classified as resolved.

**Figure 3:**
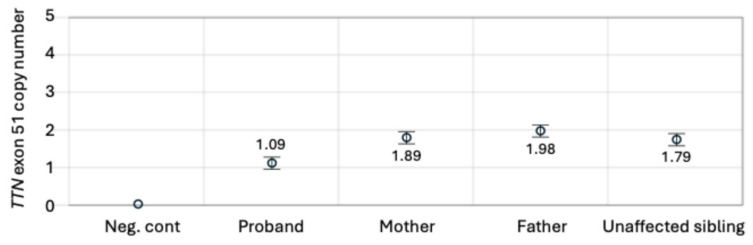
ddPCR confirmation of de novo *TTN* deletion in BOS_1210. Droplet digital PCR (ddPCR) analysis for exon 51 in the proband and family members validated the de novo nature of the heterozygous deletion of *TTN* exons initially detected by genome sequencing and confirmed by RNA evidence of abnormal splicing. This orthogonal confirmation provides high-confidence evidence for the pathogenic structural variant underlying the congenital fiber type disproportion phenotype in BOS_1210.

**Figure 4:**
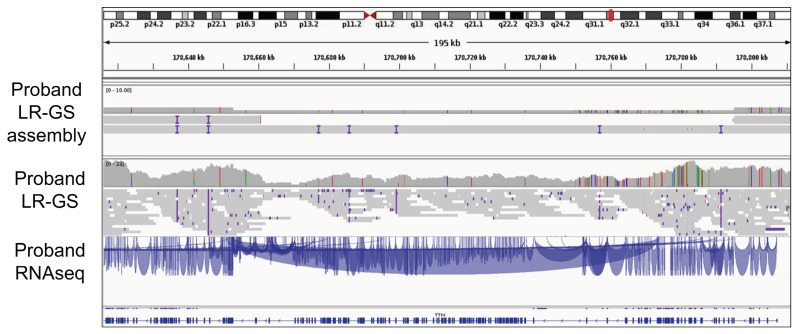
Multimodal genomic analysis of the titin (*TTN*) gene in a proband with sporadic CFTD. IGV view of skeletal muscle short read RNAseq (bottom track) shows normal splicing pattern with one large splice event skipping exons 34-200 (exon map at bottom with the gene oriented on the antisense strand from right to left). LR-GS (middle track) shows reduction in read depth in region of the exon skip and lack of heterozygosity for any rare variants in this region, indicated by solid red (T), blue (C), green (A) or orange (G) lines. An assembly of these long reads (top track) confirms a sharp drop at boundaries of this deletion and analysis of junction spanning reads confirmed the breakpoints defining the deletion as chr2:178,652,765–178,772,147del.

BOS_1210: A large deletion within the titin (*TTN*) gene was identified by next generation panel testing and proved to be de novo by ddPCR (Figure 3) [7]. To confirm the event, define the breakpoints, and determine parental origin, HiFi reads were assembled with hifiasm v0.18.9 and compared with RNAseq results of skeletal muscle RNA (Figure 4) and the deletion was proven to encompass exons 34–200 in *TTN* (chr2:178,652,765–178,772,147; 119,383 bp). The automatic assembly did not traverse this region because of a local graph ambiguity in a repetitive segment. Manual intervention was restricted to removal of three reads that created spurious short overlaps inconsistent with the surrounding haplotype structure, after which a locally consistent haplotype-resolved assembly was obtained and the deletion was assigned to the paternal haplotype (top track in Figure 4). 

The maternal haplotype did not contain the approximately 119 kb deletion. It contained 16 coding SNPs as well as a known 270 bp VNTR insertion (nine copies of a 30 bp repeat unit) in intron 45. Together with the abnormal RNA splicing pattern and prior ddPCR confirmation, these data established the de novo heterozygous *TTN* deletion as the molecular diagnosis. Multiscore ranked *TTN* as the top gene tested (rank = 100%). This result has been subsequently reported by Perrin et al. [7]. The family was therefore classified as resolved.

MAN_1875: After a year of systematic review across technologies, no additional candidate variants of clear relevance were identified for this family. Reanalysis therefore focused on the previously reported de novo cysteine-substituting *NOTCH3* variant (chr19-15170764 A>G, NM_000435.3:c.4798T>C, p.Cys1600Arg) previously classified as a VUS. Although cysteine-altering *NOTCH3* variants are an established mechanism in CADASIL and have occasionally been reported with peripheral neuropathy and optic neuritis, the proband’s broader phenotype, including recurrent infections and thyroid carcinoma, was not typical for classic *NOTCH3*-related small-vessel disease. No cerebrovascular phenotype suggestive of classic CADASIL was documented in the available records. However, recent evidence implicates gain-of-function *NOTCH3* variants in familial partial lipodystrophy [16], providing a plausible explanation for the proband’s lipodystrophy-like changes and hepatic steatosis/hepatosplenomegaly. We therefore regarded *NOTCH3* as a possible partial molecular explanation, but the family remained unresolved overall. Multiscore ranked the phenotype match between the proband and *NOTCH3* in the top 0.6% of genes tested (rank = 99.4%).

MAN_0009: SR-GS revealed a de novo *UBA6* splice-region variant, chr4-67624989 C>A, NM_018227.6:c.2712+5G>T, not seen in population databases. This variant was also identified on LR-GS. Pangolin scored the variant at 0.32 for splice loss and 0.34 for splice gain, suggesting that a major splicing effect was unlikely. Iterative reanalysis of the existing SR-GS data, informed by updated disease gene knowledge rather than a new sequencing modality alone, subsequently identified that the individual carried a recurrent pathogenic insertion in the U4 snRNA gene *RNU4-2*, known to cause monogenic intellectual disability (NR_003137.3(*RNU4-2*):n.64_65insT) [17]. This established a molecular diagnosis for the proband. The Multiscore phenotype match between the proband and *RNU4-2* was in the top 8.9% of genes tested (rank = 91.1%), in part because only one prior observation was present in the GeneDx knowledgebase at the time; within that context, this remained a strong prioritization result.

MAN_0146: Biallelic *NARS2* variants were identified via trio SR-GS (maternal: chr11-78561933 C>T, NM_024678.6:c.514-2314G>A; paternal: chr11-78571428 AC>A, NM_024678.6:c.157del, p.Val53Serfs*12). The maternal deep-intronic variant had a SpliceAI score of 0.49, suggesting a possible splicing effect. The affected sibling (MAN_0146-04), who died of a similar condition, also carried both variants, and both variants were detected on long-read sequencing. RNA-seq from cardiac tissue did not show an impact on splicing for the maternal variant; therefore, this family was retained as a strong candidate diagnosis rather than a functionally confirmed or fully resolved finding. The Multiscore phenotype match between the proband and *NARS2* was in the top 5.8% of genes tested (rank = 94.2%).

MAN_2064: Multimodal analysis identified a 1324 bp heterozygous deletion in ZEB2 (chr2:144,396,594–144,397,918) in both brothers, detected by LR-GS. Reinspection of the SR-GS data confirmed that the deletion had been present but previously unrecognized. Manual inspection of the parental data did not identify the deletion in either parent, raising the possibility of gonadal mosaicism. Given the established role of ZEB2 haploinsufficiency in Mowat–Wilson syndrome [18] and the concordant developmental and craniofacial features observed in these siblings, this finding provides a strong candidate explanation for their condition. However, because the event was an intragenic deletion in the setting of an atypical adult presentation and lacked RNA-based confirmation, we classified the family as a strong candidate rather than definitively resolved within this study. The Multiscore phenotype match between the proband and ZEB2 was in the top 2.7% of genes tested (rank = 97.3%). No additional contributory variants were identified through OGM, RNA-seq, or mobile-element analysis. xTea did not identify mobile elements of interest in any family.

## 4. Discussion

Using a coordinated multimodal framework, we resolved three of six families and generated strong candidate findings in two more. Because this was a small, highly selected pilot cohort, these proportions should be interpreted descriptively rather than as stable estimates of diagnostic yield; the corresponding Wilson 95% confidence interval for the resolved proportion is wide (approximately 19–81%). Representative diagnostic yields from related published cohorts are summarized in Table 6.

The principal contribution of this study is therefore not a precise yield estimate, but a real-world demonstration of how repeated phenotype review, reanalysis of existing short-read data, and selective escalation to orthogonal assays can be organized within a weekly interdisciplinary workflow for deeply investigated families. Thus, older short-read exome or genome datasets should not be viewed as static negative tests; rather, they represent reusable diagnostic resources whose value can increase over time as computational pipelines, disease gene knowledge, and complementary multimodal technologies mature.

The cases also illustrate that the added value was heterogeneous rather than attributable to any single technology. BOS_1596 was resolved by integrating re-reviewed genome data with RNA-seq evidence of monoallelic expression. BOS_1210 depended on structural-variant detection in prior short-read data, but LR-GS added breakpoint resolution and parental phasing, while RNA evidence supported pathogenicity. MAN_0009 was solved chiefly through iterative reanalysis of existing SR-GS in light of newly recognized disease biology (*RNU4-2*), underscoring that the multimodal framework includes knowledge reinterpretation and not only new data generation. MAN_0146 and MAN_2064 demonstrate how multimodal follow-up can elevate plausible candidates without necessarily reaching a definitive diagnosis.

Only MAN_1875 yielded no definitive new molecular finding from multimodal sequencing; however, continued literature surveillance enabled reinterpretation of a previously reported de novo *NOTCH3* variant in light of emerging evidence linking gain-of-function *NOTCH3* variants to familial partial lipodystrophy [16]. This underscores that even when additional technologies are unrevealing, periodic reinterpretation of existing variants can yield clinically meaningful insight. At the same time, the case remained unresolved overall, emphasizing the importance of distinguishing partial molecular explanations from fully reportable diagnoses.

Challenges remain in scaling multimodal sequencing for broader clinical use. Cost, infrastructure demands, bioinformatics complexity, and data-integration hurdles are important barriers, and equitable access must be prioritized. Technical limitations were also evident in this cohort. The putative NEB deletion detected on LR-GS was not confirmed by OGM and was reclassified as a false positive, emphasizing that structural calls in repetitive or low-complexity regions require orthogonal confirmation. Conversely, the *TTN* deletion required manual local assembly resolution because automatic assembly failed in a complex region. RNA-seq was informative only when disease-relevant tissue was available and may miss context-specific splicing or expression effects, as illustrated by the absence of a detectable *NARS2* splicing abnormality in available cardiac tissue. OGM and mitochondrial analyses were complementary rather than uniformly informative. These limitations argue for modality-specific quality control, explicit escalation criteria, and cautious interpretation of apparently concordant findings.

In summary, systematic application of multiple sequencing modalities within a coordinated framework can improve case resolution in real-world unsolved rare-disease families, but the benefit derives from the combination of technologies, updated knowledge, and structured team interpretation rather than from any single assay. Multiscore was useful for prioritizing phenotype-consistent genes, especially in cases such as *TTN*, *KLHL40*, and *ZEB2*, but it functioned as a triage tool rather than a diagnostic arbiter and was limited by the gene content of the active MDR. Larger prospective studies will be needed to determine when multimodal escalation adds value beyond short-read reanalysis alone and to define the most efficient pathways for clinical implementation. Even in its current form, the workflow described here provides a practical model for integrating reanalysis, orthogonal sequencing, and multidisciplinary review in rare disease genomics.

## Figures and Tables

**Table 1:** Overview of participating families and probands. Summary of the six families included in this study, with the cohort/family identifier, proband age and sex, and an abbreviated description of the major clinical features. Two families included more than one affected individual (MAN_0146 and MAN_2064), one included an unaffected sibling (BOS_1210), and the remaining families were simplex.

Cohort/Family ID	Age and Sex	Abbreviated Phenotype
MAN_0009	29 yo male	dysmorphisms, microcephaly, severe cognitive impairment, abnormal movements, bilateral sensorineural hearing loss, visual impairment, nystagmus
MAN_1875	19 yo male	demyelinating neuropathy with muscle neurogenic atrophy, hepatic steatosis, lymphedema
BOS_1210	20 yo male	history of arthrogryposis multiplex, clinicopathological diagnosis of congenital fiber type disproportion
BOS_1596	4 yo male	clinicopathological diagnosis of nemaline myopathy, hypotonia, proximal muscle weakness, global delay
MAN_2064	two male siblings in 40/50s	ID, hypospadias, connective tissue disorder, keratoconus, dysmorphisms
MAN_0146	two female siblings, neonates	deceased, cardiomyopathy

**Table 2:** Sequencing and analysis modalities applied to each family. Summary of the multimodal technologies performed across the six participating families. Columns indicate whether PacBio (Menlo Park, CA, USA) Revio LR-GS (probands only), Bionano Technologies (San Diego, CA, USA) Saphyr OGM, SR-GS, LR-GS with PacBio Sequel IIe, RNA-seq, and targeted mitochondrial genome sequencing performed by GeneDx (Gaithersburg, MD, USA) were carried out. An ‘x’ denotes that the modality was performed for the indicated family.

Family	Revio LR-GS (Probands Only)	Bionano Saphyr OGM	Short-Read GS	Sequel IIe LR-GS	GeneDx Mito	RNA-Seq
BOS_1596		x	x	x	x	x
BOS_1210			x	x	x	x
MAN_1875	x		x	x	x	x
MAN_0009	x		x	x	x	x
MAN_0146	x		x	x	x	x
MAN_2064	x		x	x	x	x

**Table 3:** Multiscore data informing scoring and list of genes scored for MDR1 and MDR2.

Metric	MDR1	MDR2
GDx case date	31 December 2023	30 September 2025
Literature date	31 December 2023	30 September 2025
OMIM release	3 September 2023	1 September 2025
size of gene list	19,215	19,260
gene list contains all mRNA genes (GDx database)	True based on 1 August 2023 data	True based on 28 August 2025 data
gene list contains all validated and candidate diseases (GDx database)	False	True based on 28 August 2025 data
gene list contains noncoding genes requested by clinical team	False	True based on 28 August 2025 data

**Table 4:** Multiscore results for the genes of interest in participant families and probands. GDx cc refers to the number of cases with a monogenic positive report on a given gene at the time the MDR was collected.

Case ID	Gene HGNC ID	Gene Symbol	Multiscore Rank	GDx cc	Multiscore Data Reference (MDR)
BOS_1210-01	HGNC:12403	*TTN*	100.0%	66	MDR1
BOS_1596-01	HGNC:30372	*KLHL40*	99.6%	4	MDR1
MAN_0009-01	HGNC:25581	*UBA6*	83.4%	0	MDR1
MAN_0009-01	HGNC:10193	*RNU4-2*	91.1%	1	MDR2
MAN_0146-01	HGNC:26274	*NARS2*	94.2%	5	MDR1
MAN_1875-01	HGNC:7883	*NOTCH3*	99.4%	23	MDR1
MAN_2064-01	HGNC:14881	*ZEB2*	97.3%	126	MDR1

**Table 5:** Summary of results across sequencing and analytical modalities for each kindred. For each proband, the contribution of mitochondrial DNA (mtDNA) analysis, short tandem repeat (STR) analysis, mobile-element detection with xTea, short-read genome sequencing (SR-GS), Sequel IIe LR-GS, RNA sequencing (RNA-seq), and PacBio Revio LR-GS are shown. “–” indicates that no unique new causal finding or no contributory variants were identified, “+” indicates supportive evidence for a candidate variant, and “np” indicates that the modality was not performed. Where applicable, the candidate gene(s), variant type(s), and genomic coordinates (GRCh38) are reported. This table highlights the complementary yield of different modalities and illustrates which approaches provided supportive evidence toward case resolution.

Kindred	mtDNA	STR	xTEA Mobile DNA	SR-GS SNP/CNV	Sequel IIe LR-GS SV/CNV	RNA-Seq	Revio LR-GS
BOS_1596	–	–	–	*KLHL40*, compound heterozygous variants maternal: chr3-42692145G>T, NM_152393.4:c.Ter152G>T paternal: chr3-42686291 C>T, NM_152393.4:c.673C>T (p.Arg225Cys)	–	+	np
BOS_1210	–	–	–	*TTN* del chr2:[178652765_178772147del];[=] NM_001267550.2(*TTN*): c.[(7855+1_7856-1)_(38875+1_38876-1)del];[=]	*TTN* del chr2:[178652765_178772147del];[=] NM_001267550.2(*TTN*): c.[(7855+1_7856-1)_(38875+1_38876-1)del];[=]	+	np
MAN_1875	–	–	–	*NOTCH3*, de novo missense (VUS; possible familial partial lipodystrophy association) chr19-15170764 A>G (NM_000435.3:c.4798T>C, p.Cys1600Arg)	–	–	–
MAN_0009	–	–	–	*RNU4-2*, recurrent de novo NR_003137.3(*RNU4-2*):n.64_65insT	–	–	–
MAN_0146	–	–	–	*NARS2*, compound het (frameshift + predicted splice) maternal: chr11-78561933 C>T, NM_024678.6:c.514-2314G>A paternal: chr11-78571428 AC>A, NM_024678.6:c.157del, p.Val53Serfs*12	–	–	–
MAN_2064	–	–	–	Retrospective confirmation of *ZEB2* del chr2:144,396,594–144,397,918;[=]	*ZEB2* del chr2:144,396,594–144,397,918;[=]	–	–

**Table 6:** Diagnostic yields across published cohorts using genome sequencing, long-read sequencing, reanalysis, and multimodal approaches. Comparison of cohort sizes, methodologies, and reported yields from representative studies of unresolved rare-disease cases. Because cohort definitions, prior testing, and outcome criteria differ substantially across studies, these values are descriptive and are not intended for direct statistical comparison. The current pilot study (N = 6) achieved a 50% resolution rate, but with a wide Wilson 95% confidence interval (approximately 19–81%) due to small sample size.

Study	Modality/Framework	Cohort Size (N)	Selection/Setting	Reported Yield	Notes
Wojcik et al. [3]	Short-read genome sequencing in clinical rare disease	822 families total: 744 initial-cohort families and 78 replication-cohort families	Broad clinical indications, multi-site	Molecular diagnosis in 218/744 initial-cohort families (29.3%); diagnoses requiring GS specifically in 61/744 families (8.2%)	Large, generalizable cohort with primarily SR-GS
Negi et al. [4]	Long-read (nanopore) genome assembly + variant calling in previously unsolved cases	41 families, 98 samples total	Post-negative SR-GS enrichment	Diagnostic variants established by long-read sequencing in 11 probands (27%)	Larger cohort demonstrating a sizeable yield in diagnostic rate after LRS
Sanford Kobayashi et al. [5]	Long-read sequencing in clinical setting	35 samples total: 30 undiagnosed subjects from 26 families plus 5 controls	Post-SR-GS	1/30 (3.3%) new diagnoses among undiagnosed subjects	Underscores value of targeted multimodal use.
Fung et al. [1]	Iterative reanalysis (ES)	104 individuals in the initial ES cohort; 46 undiagnosed individuals underwent reanalysis.	Longitudinal follow-up	Initial diagnostic yield 43/104 (41%); reanalysis added 12 diagnoses among 46 reanalyzed individuals. Overall yield increased to at least 55/104 (≥53%)	Reanalysis added to diagnostic yield; 72.2% of diagnosed individuals had a management change.
Schmitz-Abe et al. [6]	Bioinformatic reanalysis of clinical exomes	102 probands: 74 CES-negative cases and 28 cases with candidate variants; yield assessed in 75 CES-negative/reclassified cases	Previously tested, unsolved	24/75 (32.0%) confirmed or potential genetic diagnoses, including 6 known disease gene diagnoses and 18 candidate-gene findings	Demonstrates reanalysis benefit
CLARITY Challenge [19]	Team-based interpretation process	3 families; 12 individuals with WES and 10 with WGS	Competition; multi-team	Process-focused, (2 out 3 families considered solved)	Supports interdisciplinary review improving diagnostic outcomes.
Current study	Multimodal integration (SR-/LR-GS, RNA-seq, OGM, mobile-element scan) + weekly interdisciplinary review	6 families	Post-negative SR-ES/GS; real-world unsolved	50% resolved (3/6); 33% strong candidates	Proof-of-concept; small N with Wilson 95% CI ≈ 19–81% for the resolved proportion; demonstrates value of coordinated multimodality.

## Data Availability

All data that support the findings of this study are available on request to the corresponding author. Sequencing data will be deposited in dbGaP (accession TBD) on publication.

## Supplementary Materials

The following supporting information can be downloaded at https://www.mdpi.com/article/10.3390/genes17060647/s1, Figure S1: Mean coverage across LR and SR GS; Figure S2: Simplified Pedigrees.
