# Supplementary material for: Multimodal Sequencing and Reanalysis Approaches to End the Diagnostic Odyssey of Individuals with Suspected Rare Monogenic Diseases
Source: Genes (Basel). 2026 May 31;17(6):647. doi: 10.3390/genes17060647 (PMC13299716; doi:10.3390/genes17060647)
Supplement: Supplementary file 1 [file genes-4204307-supplementary.pdf › genes-4204307-supplementary.pdf]

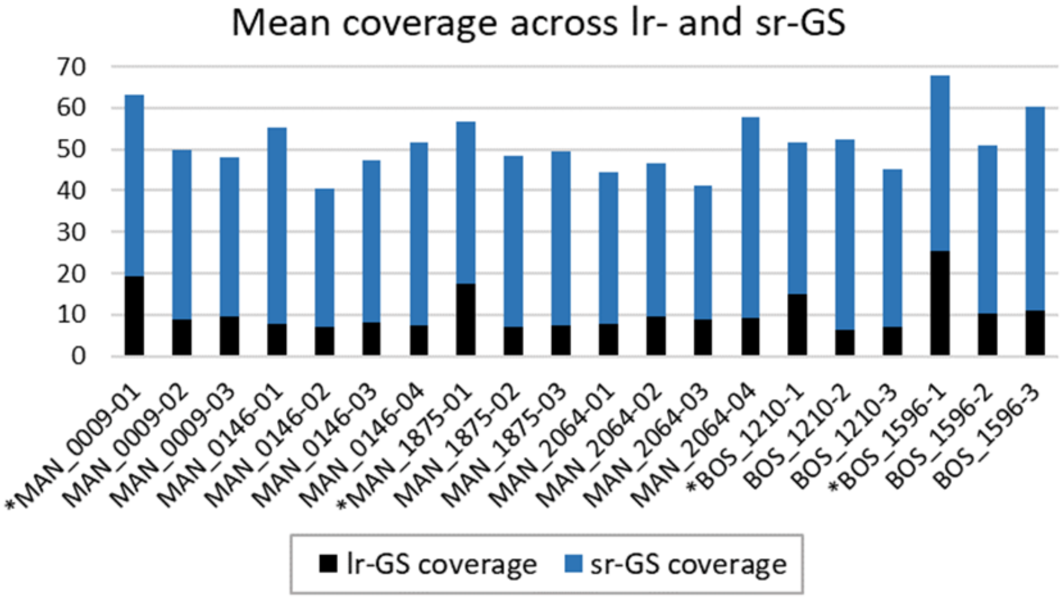

Supplemental Figure S1. Sequencing quality metrics for short- and long-read genome sequencing. Summary of quality control metrics for short-read (SR-GS) and long-read (LR-GS) genome sequenc-ing across the study cohort. Metrics include overall coverage, read-length distributions, and error profiles, demonstrating that both sequencing approaches achieved high-quality datasets suitable for structural and single-nucleotide variant detection.

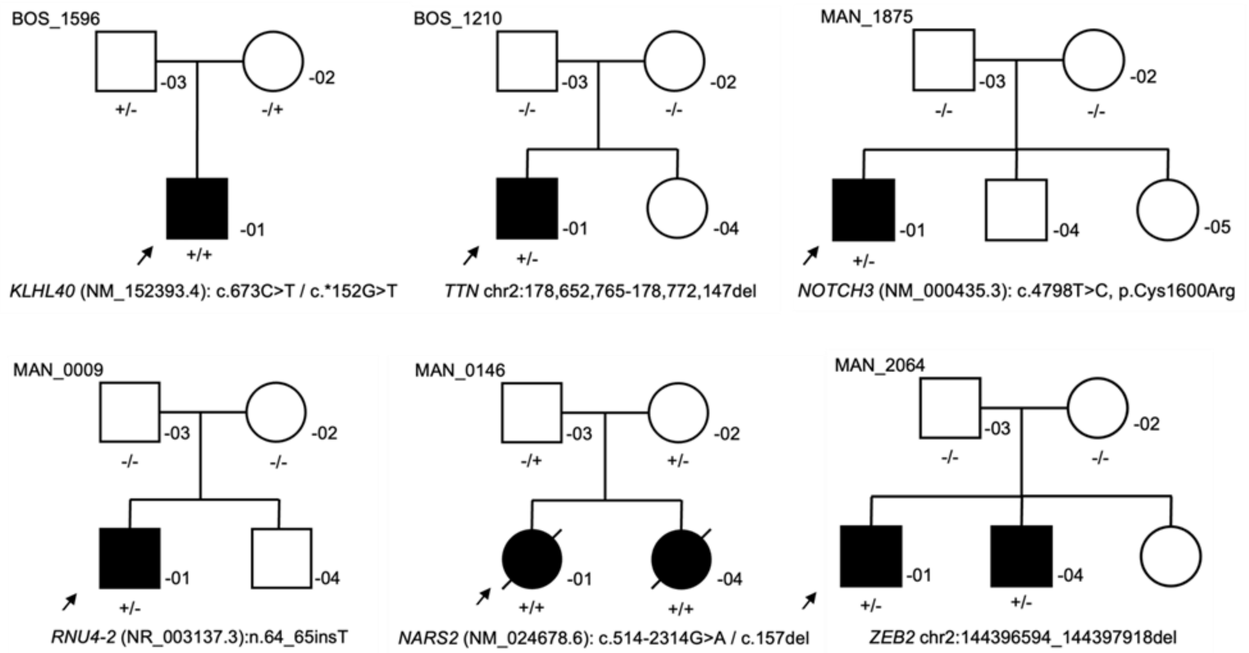

Supplemental Figure S2. Simplified pedigrees of participating families. Simplified pedigrees for each kindred analyzed in the manuscript. Filled symbols indicate affected individuals, a diagonal slash indicates deceased individuals, and arrows indicate probands. Indi-vidual IDs are shown to the right of each symbol and genotypes indicated below.
